# Vital capacity evolution in patients treated with the CMCR brace: statistical analysis of 90 scoliotic patients treated with the CMCR brace

**DOI:** 10.1186/1748-7161-6-19

**Published:** 2011-08-31

**Authors:** Jean-Claude Bernard, Julie Deceuninck, Céline Kohn

**Affiliations:** 1 Croix Rouge française - Centre Médico-Chirurgical de Réadaptation des Massues Children and adolescents Physical and Rehabilitation Medicine Department 92, rue Edmond Locard - 69322 Lyon Cedex 05 - France

**Keywords:** scoliosis, respiratory capacity, brace with mobile pads

## Abstract

**Summary:**

**Objective**

To study the evolution of pulmonary capacity during orthopaedic treatment of scoliosis with the CMCR brace.

**Background**

Investigating the impact of moderate scoliosis on respiratory capacity and its evolution during CMCR brace treatment with mobile pads.

**Context**

Several studies demonstrate the impact of scoliosis on respiratory capacity but few of them focus on the impact of bracing treatment. We studied the evolution of the pulmonary capacity of a cohort of 90 scoliotic patients.

**Methods**

This retrospective study included 90 scoliotic patients treated since 1999 with a brace with mobile pads called CMCR (n = 90; mean age: 13 years; 10-16). These patients were diagnosed with an idiopathic scoliosis (mean angulation 20.6°). All patients underwent a radiographic and respiratory evaluation at the beginning, the middle and the end of treatment.

**Results**

*Mean age at treatment start was *13. Before treatment, our patients did not have a normal pulmonary capacity: Forced Vital Capacity (FVC) was only 75% of the theoretical value. All curvature types (thoracic, thoraco-lumbar and combined scoliosis) involved this reduced pulmonary capacity, with moderate-angulated scoliosis having a negative impact. At the beginning of brace treatment, the loss of real vital capacity with brace (0.3 litres) was 10% lower than without brace.

At CMCR removal, the FVC had increased by 0.4 litre (21% +/- 4.2% compared to the initial value). The theoretical value had increased by 3%. This positive evolution was most important in girls at a low Risser stage (0,1,2), and before 11 years of age.

**Conclusion**

These results supported our approach of orthesis conception for adolescent idiopathic scoliosis which uses braces with mobile pads to preserve thorax and spine mobility.

## Introduction

Scoliosis is a three-dimensional deformity of the spine, which leads to a torsion of several vertebrae causing a thoracic deformity [1] and a reduction of cardiac and pulmonary capacity [2]. Until quite recently, only two kinds of treatment were considered efficient: spinal surgery and brace treatment [3-7]. In 2003, Weiss showed that intensive rehabilitation could reduce curvature progression [8], even if in the meantime Negrini was more cautious [9]. In a 2008 literature review Negrini and al. finally demonstrated the efficacy of exercises in reducing the progression rate and/or improving the Cobb angles [10]. Our rehabilitation programms meet the guidelines defined in 2008 by the SOSORT Consensus [11]. According to these criteria, the aim of brace treatment is to correct spinal deformities and limit their evolution during the growth period. Brace treatment is still associated with physical exercises, at a rate of one physiotherapy session per week in addition to home exercises, to improve the efficacy of bracing and prevent postural collapse [12].

**Figure 1:**
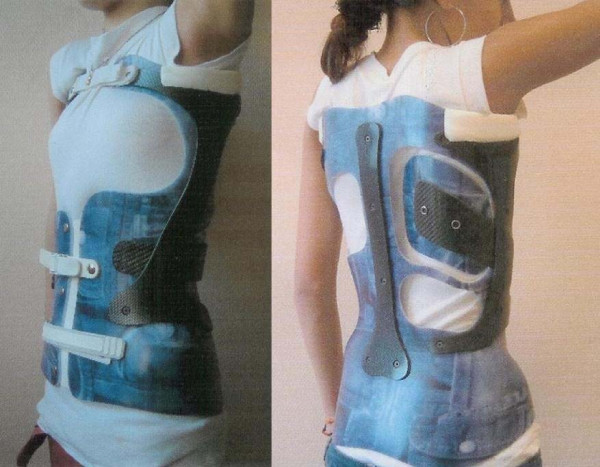
**CMCR brace, ¾ anterior view and ¾ posterior view**.

The currently used braces have proved to be really efficient [13] orare promising [3,14]. However, they still present disadvantages because of their rigidity, especially as they limit the thoracic function because of the lack of expansion while breathing in [15]. For Lacheretz, [16,17], the radiocinematographic study of scoliotic patients shows disturbed ventilatory kinetics especially regarding costal movements of the hollow side and decreased motility of the corresponding diaphragmatic hemi-dome. In order to minimize or eliminate these disadvantages, a new brace was designed [18,19], with pads located on the humps to retain some mobility. The features of this brace named « Corset Monocoque Carbone Respectant la Respiration » (carbon brace preserving lung capacity, or CMCR brace) is detailed in this study (Figure 1).

We have then study the evolution of the respiratory capacity in a group of scoliotic patients undergoing orthopaedic treatment during the growth period, in order to analyse the brace impact on the development of pulmonary capacity. We compared measures at baseline, at the beginning of brace treatment, and at weaning. We have specify which variables have an impact on FVC. The results of this study were pre-released in a 2005 publication [18], when some patients had not completed their treatment yet.

The objective of this study was to investigate the evolution of the pulmonary capacity during the orthopaedic treatment of scoliosis with the CMCR brace.

## Materials and methods

### Patients

Our study included 90 patients treated since 1999. They were enrolled in this study at the beginning of their treatment with a mean age of 13 years for girls and 13 years and 4 months for boys (Table 1). The mean age at brace removal for this cohort of patients having completed the treatment was 15 years and10 months for the 87 girls and 15 years and 8 months for the 3 boys. All scolioses were idiopathic (neurological examination was negative), and progressive (clinical and radiological deterioration between two successive examinations (6 months) during the growth period). Malformative, neurological or dysplastic scolioses were excluded from this study. Lumbar scolioses are usually treated with another kind of brace, and thus were not included either.

**Table 1:** Characteristics of the population at the beginning of the treatment by CMCR brace and at the definitive brace removal

Variable	Group at the beginning of treatment		Group at the definitive brace removal	
**Gender**	Girls	87 (97%)	Girls	87 (97%)
	Boys	3 (3%)	Boys	3 (3%)

**Mean age**	Girls	13 years	Girls	15 years 10 months
	Boys	13 years 4 months	Boys	15 years 8 months

**Curvative type**	Double-major	67 (74.4%)	Double-major	67 (77.0%)
	Lumbar	3 (3.3%)	Lumbar	-
	Thoracic	7 (7.8%)	Thoracic	7 (8.0%)
	Thoraco- lumbar	13 (14.4%)	Thoraco-lumbar	13 (14.9%)

**Cobb angle**	Double-major	20.9° 20,0°	Double-major	20.6° 16.9°
	Thoracic	20.3°	Thoracic	17.3°
	Thoraco- lumbar	24.1°	Thoraco- lumbar	21.5°

**Skeletal** **Maturity**	Risser 0	33 (36.7%)	Achieved	
	Risser 1	15 (16.7%)		
	Risser 2	21 (23.3%)		
	Risser 3	14 (15.6%)		
	Risser 4	7 (7.8%)		

**Theoretical VC**	Girls	3.2 litres	Girls	3.6 litres
	Boys	4.1 litres	Boys	4.5 litres

**FVC**	Girls	2.3 litres	Girls	2.7 litres
	Boys	2.9 litres	Boys	4 litres

Ninety patients with an idiopathic scoliosis were selected for this study: 87 girls and 3 boys (13 years of average age) with a minimum age at the beginning of treatment of 10 years and a maximum age of 16 years.

### Instrumentation

The CMCR brace is a light brace, reinforced with carbon blades instead of the usual metallic structure, which is put on without any prior casting reduction [18]. The corrective effect of this brace is achieved by the addition of mobile pads on the humps, which are associated with opposite supports to make the key pressure efficient and to provide a satisfying trunk balance. These pads are accurately positioned and adjusted with the help of a three-dimensional analysis [1,20] instead of a simple front radiography.

The specific features of this brace can be summarised as follows: the adjustment of the device yields many possibilities for the orientation of pressure forces above the humps; its mobile pads preserve trunk kinetics by the use of pre-stressed carbon (which returns to its original shape). In short, it is a single-bodied brace, light and easy to put on by the child or teenager himself.

### Methods

Data was collected including several variables: age, gender, kind of scoliosis, Risser stage, Cobb angle measured on a front radiograph in standing position, standing height, weight, theoretical vital capacity [11], forced vital capacity, ratio between forced vital capacity/theoretical vital capacity.

**Figure 2:**
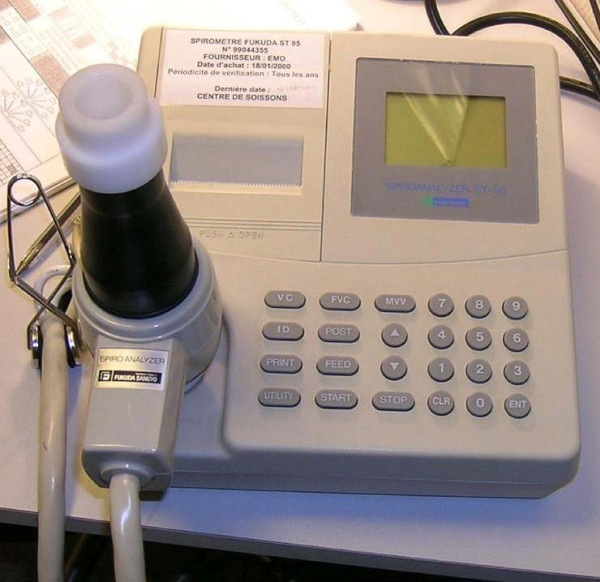
**Electronic spirometer Spirolyser SPL 100**.

The forced vital capacity (FVC) or respiratory capacity is the maximal quantity of air that can be breathed out and is measured with a spirometer (Figure 2).

The FVC is the sum of current volume (CV), reserve inspiratory volume (RIV) and reserve expiratory volume (REV). The patients are evaluated in a sitting position and are instructed to breathe into the spirometer as deeply and quickly as possible. This test should be performed three times and only the best measure will be kept. The FVC was systematically compared to the theoretical vital capacity. The theoretical value is calculated automatically by the spirometer based on age, gender and cutaneous surface. Besides, it is contingent on weight and height according to standard ZAPLETAL [21]. At the beginning of the treatment, when the brace is well tolerated, a measure of breathing capacity is achieved with the brace on in the same conditions. Trunk casting was completed with computer assistance by Orten^®^[22,23].

All patients received physiotherapy during orthopedic treatment at a rate of one session each week from a private physiotherapist. It is advisable for each patient to repeat the exercises at home several times a week. During the first year of orthopaedic treatment, the physiotherapy sessions are carried out with the brace on.

### Statistical analysis

Statistical analyses were made with software SPSS 16.0. Data about CMCR included some missing values, which had to be taken into consideration for the data analysis.

The reduction in the curvature and changes in vital capacity between the various stages of treatment were studied using the Student test for comparison between 2 parameters and two-ways ANOVA tests to compare more than two parameters, after checking the normality (Shapiro-Wilk) and homogeneity of variances (Levene's test). If any of these conditions were not valid, the nonparametric Mann-Whitney (to compare two independent samples) and Kruskal-Wallis (to compare more than two independent samples) tests were used instead.

A significant difference was defined when the *p *-value < 0.05, and results were given with a 95% confidence interval.

## Results

### 1 -Beginning of treatment

Combined scoliosis (double-major curved) was the most frequent: more than 74% of patients (Table 1). More than 76% of the patients began the treatment before Risser 3. The mean scoliosis angulation was 20.6°, depending on the type of curvature. The mean forced vital capacity (FVC) was 2.4 litres. The mean FVC was only 75% of the theoretical value.

### 2 - Evolution of forced vital capacity and influent variables at the CMCR setting up

#### 2.1 - FVC at the beginning of orthopaedic treatment, with and without the brace

At the beginning of orthopaedic treatment, the paired Student test highlighted a significant difference (*p *= 0.000) between FVC without brace (2.4 litres) and FVC with brace (2.1 litres): with an average FVC reduction of 0.3 ± 0.03 litre (corresponding to 12 ± 1.2% of the FVC without a brace).

#### 2.2- FVC at the beginning of brace treatment compared to the theoretical value

Before brace treatment, the mean FVC was 75% of the theoretical value, and was reduced to 62.5% of the theoretical value with the brace on at the beginning of the treatment. There was therefore a loss of 10 ± 1% of vital capacity compared to the theoretical vital capacity.

#### 2.3 - Variables impact on real respiratory capacity decrease at the beginning of brace treatment

Neither gender, curvature type, nor Cobb angle observed at baseline was found to have any influence on the FVC decrease at the brace setting up (Table 2).

**Table 2:** Impact of variables (gender, prior casting, kind of scoliosis, Risser stage, age at the beginning of treatment, Cobb angle at the beginning of treatment) on real respiratory capacity decrease at the beginning of brace treatment (FVC evolution in litres) and on the difference between the percentage to the theoretical vital capacity without and with CMCR brace.

			FVC evolution (in litres)		Evolution in percentage of the VC (%)	
**Variables**	**Groups**	**N**	**Mean or** **Median**	**P-value**	**Mean**	**P-value**

Gender (**)	Girls	87	0.30 L	0.842	9.6	0.773
	Boys	3	0.30 L		8	

**Kind of scoliosis** (**)	Double-major scoliosis Thoraco-lumbar	67	0.20 L		7.8	
	scoliosis	13	0.30 L	0.138	10	0.122
	Thoracic scoliosis	7	0.30 L		13	

Risser stage at the beginning of treatment (**)	Patients Risser stage 0, 1 or 2	69	0.30 L	0.055*	10.4	0.097
	Patients Risser stage 3 or 4	21	0.20 L		6.6	

Age at the beginning of treatment (**)	Patients ≤ 11 years	12	0.30 L	0.037*	12.8	0.173
	Patients > 11 years	78	0.20 L		9	

**Cobb angle at the beginning of treatment**	Patients curve < 30°	84	0.26 L	0.31	9.5	1
	Patients curve ≥ 30°	6	0.35 L		9.5	

(*)p-value < 0.05, which is significant

(**)a non parametric test was used; in that case, medians (and not means) are presented.

Patients at Risser stage 3 or below had a significantly greater decrease of FVC than those with a Risser stage above 3 at brace setting up.

Similarly, we note that patients under 11 have a greater decrease of FVC than those over 11.

#### 2.4 - Impact of variables on the difference between the percentage of FVC measure and the theoretical vital capacity with and without CMCR brace

There was no significant difference between the percentage of FVC measure ant the theorical value (Table 2).

### 3 -Treatment outcome

The characteristics of the CMCR group at the definitive brace removal are detailed in Table 1.

By the end of our study, 90 patients had completed their treatment with the CMCR brace. The mean age of our patients was 13, and the mean angulation was 20.6° with variations depending on the curvature type. The actual FVC was 2.7 litres in girls, boys having a better FVC than girls. The mean theoretical vital capacity was 3.6 litres.

### 4 - Evolution of vital capacity and influent variables at brace weaning

A paired Student *t *test highlighted a significant difference (*p *= 0.000) between FVC before bracing (2.3 litres) and at weaning (2.7 litres): the mean increase was 0.04 ± 0.1 litre, which corresponds to an increase of about 21% ± 4.2% of FVC. The treatment of scoliosis with the CMCR brace therefore increased the FVC, compared to the theoretical vital capacity (Table 1).

We looked into variables that might have a significant effect on FVC increase. (Table 3)

**Table 3:** Influent variables on FVC increase compared to the theoretical vital capacity during the CMCR treatment.

Indicator	Variables	Groups	N	Mean or median	P-value
**FVC evolution** **(in litres)**	**Gender**	Girls	87	-0,4	0,011*
		Boys	3	-1,4	
	
	**Prior casting?**	Yes	5	-0,3	0,784
		No	85	-0,4	
	
	**Kind of scoliosis**	Double-major scoliosis	67	-0,3	0,408
		Thoraco-lumbar Scoliosis	13	-0,7	
		Thoracic scoliosis	7	-0,5	
	
	**Risser stage at the beginning of treatment**	Patients Risser stage 0, 1 or 2	69	-0,5	0,001*
		Patients Risser 3 or 4	21	0	
	
	**Age at the beginning of treatment**	Patients ≤ 11 years	12	-0,8	0,011*
		Patients > 11 years	78	-0,3	
	
	**Cobb angle at the beginning of treatment**	Patients curve < 30°	84	-0,4	0,439
		Patients curve ≥ 30°	6	-0,1	

(*) means that p-value < 0.05, which is significant

*- **Gender **(p = 0,011): *the increase was most important in boys (positive difference of 1.4 ± 0.4 litre), however only 3 boys were included in our study.

*- **Risser **at the beginning of treatment (difference of 0.5 *± *0.2 litre; p = 0,001): *the lower the Risser stage was, the more the respiratory capacity increased at the end of treatment.

*- **Age **at the beginning of treatment (difference of 0.8 *± *0.3 litre; p = 0,011): *The younger the patient was at the beginning of the treatment the more the respiratory capacity was increased at the end.

These results showed that the FVC seemed to increase most when treatment began early, without negative influence on growth of the thorax.

## Discussion

Even if our programmes met the criteria published in 2008 by the SOSORT [11], we were not able to follow the guidelines proposed by the SOSORT in 2006 [13], and especially the prognostic risk [24] as this study began in 1999. We use the CMCR brace to treat combined (thoracic or thoraco-lumbar) scolioses, without prior casting in 95.6% of cases (if scoliosis angulation is moderate: mean Cobb between 20° and 24,1°). Treatment is initiated as soon as the clinical and radiological examinations show a significant increase of the hump and the Cobb angle (> 5°).

Our population was similar to the one Wong studied in 2008 (randomized controlled trial) [25] to compare the effectiveness of a rigid and an elastic brace. His patients had a 20 to 30 ° Cobb angle, but lung capacity was not included in the study. The other studies we found in the literature [2,26-29] focused on smaller groups of patients, and with a greater Cobb angulation. As lumbar scoliosis patients may be successfully treated with other types of rigid or elastic braces (3-points brace, St Etienne brace), we decided to exclude them from our study [18]. In our daily practice, the choice of a brace is determined by the type of curvature, its severity, and age at the beginning of treatment [14]. In scoliosis treated with a CMCR brace, the mean reducibility for all curvature types is 42% (thoracic: 51%, thoraco-lumbar: 53%, combined: 37%).

We noticed that our patients did not have a normal FVC prior to the CMCR setting up, as it was only 75% of the theoretical value (-25% change of predicted value)[26,30].

Korovessis observed that before the introduction of the brace, this change was -11% in a comparable population treated by Boston brace and -16% in a population treated by Kennedy brace. However these two populations were different, which make their study and comparison difficult [31,32]. It would have been interesting to compare the absolute FVC values of our patients with those of a control group, rather than with the theoretical value given by the spirometer. We found a loss of 26% in girls, whereas boys were less concerned, with a 16% FVC loss, compared to the theoretical value. Barrios and al. studied a group of scoliotic teenagers (mean age 13, mean angulation 33°) [27], in which the FVC was 3.04 litres with a standard deviation of ± 0.5 litre. Barrios concluded that there was no significant difference in the basic ventilatory parameters measured in static conditions, compared to his control group of 10 girls. On the other hand, he underlined a loss in the respiratory maximal exercise tolerance test response in his scoliotic group, which could not be related to the brace. In our patients, all curvature types (thoracic, thoraco-lumbar and combined) were concerned by this FVC loss, whereas in literature only thoracic curvatures were reportedly concerned [33]. We observed that moderate-angulation scoliosis (mean Cobb between 20° and 24,1°) seems to have a negative impact on pulmonary capacity, before any conservative treatment. This might be put down to a disturbance of pulmonary physiology during the growth period (FVC loss should be considered as a scoliosis disease symptom), or to a disturbance in the biomechanics of the thoracic cage [15], originating from bone, muscle, or from an underdevelopment of the alveoli and pulmonary vasculature, encountered in patients with scoliosis [34].

Several hypotheses have been put forward to explain this disturbance: for Chu and al. [28] thorax deformity is responsible for the pulmonary impact of scoliosis, whereas disturbances of diaphragm or ribcage mobility are ruled out. For Jones and al. [26] this is due to a dysfunction between inspiratory muscles and ribcage deformity. Kotani et al. [29] found that respiratory movements are decreased in scoliotic patients, whereas Caro and Dubois [35] think that pulmonary compliance is reduced even if the ribcage is not particularly stiffened. For Giordano and al. [36] the movements of the hemi-diaphragm are reduced on the concave side. For Chu and al. [28], only patients with a severe thoracic scoliosis (from 40 to 98°) have a modified respiratory function (height of diaphragmatic domes and lung volumes while breathing in and out, explored by MRI). They found no difference between the three groups of patients (severe thoracic scoliosis, moderate thoracic scoliosis from 10 to 30°, and patients without scoliosis), whether regarding the antero-posterior and transverse thoracic diameters, or for diaphragmatic domes mobility. Chu et al. concluded that the pulmonary function is disturbed in severe scoliotic patients because lung volume is reduced on both the concave and the convex sides. Adam et al. [37] used a three-dimensional scanner to study the right - left ratios of pulmonary volumes in scoliotic patients and found a positive correlation between the increase of this ratio and the size of the hump. Pulmonary volumes are found to be correlated with the size of the hump and with the number of vertebrae included in the major curve. The shorter, the higher and the higher-rotated the major curve is, the more this pulmonary volume is decreased.

At the CMCR brace setting up, we found that the loss of real vital capacity (0.3 litre) compared to the value without a brace was 10% of the theoretical value. The higher the correction rate of the brace is (e.g. thoracic scoliosis), the greater the FVC is and the lower the correction rate is (e.g. in combined scoliosis), the lower the FVC reduction is. Refsum has shown that the vital capacity with Boston brace as well as Kennedy brace was significantly reduced to about 15% to 20% of the prebracing level [38].

In his study about the impact of bracing on the ventilatory function, Lacheretz [16,17] compared the Lyon brace to the Milwaukee brace and found only a 5% decrease of vital capacity in a group of 33 children wearing a Lyon brace. We find this result surprising in view of our experience (unpublished data, study in progress), according to which wearing a Lyon brace has much more impact on breathing than wearing a CMCR brace.

Apart from the restriction of thoracic expansion, other factors are likely to be involved in the vital capacity decrease at the brace setting up, such as abdominal compression and its consequences on diaphragmatic function. At the CMCR brace removal, the real FVC had increased of 0.5 litre, i.e. an increase of 21 ± 4.2% compared to the initial value (related to the theoretical value, this represents a 4% increase). This positive evolution is most important in girls at a low Risser stage when the treatment was started before the age of 11 (in compliance with the SRS recommendations) [39].

According to the SOSORT guidelines, the aim of conservative treatment is to stop curvature progression and improve pulmonary function [13]. We can draw the conclusion that the CMCR brace treatment does not restrain chest and lung development, even in young patients (under 11), since constraints exerted on the growing thorax preserve pulmonary and thoracic capacity.

## Conclusion

Considering the negative impact of scoliosis (even with a moderate angulation) on respiratory capacity, this capacity should be systematically and routinely measured [14] when assessing a case of scoliosis, and the loss of FVC should be considered as a scoliosis symptom. In this study, the impact on vital capacity appeared to be a symptom of the scoliotic disease rather than a consequence of the deformity itself (because of the low angles at the beginning of the treatment.).

The aim of any orthopaedic treatment is to prevent scoliosis progression, that is to say the worsening of the curves and of the impact on the breathing function and musculature. Comparisons of the reduction of the Cobb angle during the treatment clearly show that, on that point the brace is effective, but the study did not meet the criteria defined by Negrini et al., about randomised controlled trials (RCTs) or controlled clinical trials (CCTs) [14].

Although the respiratory capacity always decreases whatever the kind of brace, this decrease appears to be less important with a CMCR brace. Its "flexibility" relieves the constraints on the ribcage. And at the end of treatment with a CMCR brace, respiratory capacity had improved compared to the initial theoretical value.

Our aim is to improve the conception of our ortheses conception in order to preserve thoracic and spinal mobility, by using braces with mobile pads rather than braces with fixed pads. In the future our researches in spinal orthopaedics will be directed toward ortheses that comply with thoracic physiology (hence the importance of investigating the efficiency of elastic versus rigid braces, while systematically monitoring lung capacity [14,25]. We hope to achieve this with the use of the pre-stressed mobile pads of the CMCR brace.

## Competing interests

The authors declare that they have no competing interests.

## Authors' contributions

JC Bernard and J Deceuninck contributed equally to this work, read and approved the final manuscript. C Kohn performed the statistical analysis, and read and approved the final manuscript.

## Consent statement

Written informed consent was obtained from the patients or their parents/legal guardians for publication.
